# Death of Dr. Jonathan Cowdery

**Published:** 1852-12

**Authors:** 


DR. JONATHAN COWDERY, the oldest Surgeon in the U. S. Navy, died recently at Norfolk, aged 86 years. He had been in the service nearly fifty-three years, and took part in the Tripolitan and last war with Great Britain. In the former he was taken prisoner by the Turks and retained two years.

